# Comparing Transcatheter Mitral Valve Replacement to Transcatheter Edge‐to‐Edge Repair in Mitral Regurgitation: A Systematic Review

**DOI:** 10.1155/cdr/7522199

**Published:** 2026-09-26

**Authors:** Rama A. Safadi, Alaa Dean A. Esmail, Lauren E. DeProfio, Ahmed Adham R. Elsayed, Marc D. Basson

**Affiliations:** ^1^ College of Medicine, Northeast Ohio Medical University, Rootstown, Ohio, USA, neomed.edu; ^2^ Department of Surgery, Northeast Ohio Medical University, Rootstown, Ohio, USA, neomed.edu; ^3^ Department of Biomedical Sciences, Northeast Ohio Medical University, Rootstown, Ohio, USA, neomed.edu

**Keywords:** access site complications, hemodynamic outcomes, mitral regurgitation, MR reduction, postoperative complications, transcatheter edge-to-edge repair, transcatheter mitral valve replacement (TMVR)

## Abstract

**Background:**

Mitral regurgitation affects 2% of the population globally, with a widely increasing prevalence. Although mitral regurgitation is most commonly corrected by open surgery, there has been a recent shift toward less invasive transcatheter approaches, including both transcatheter edge‐to‐edge repair (TEER) and transcatheter mitral valve replacement (TMVR).

**Methods:**

A comprehensive literature search was conducted using four databases: PubMed, Cochrane Library, Virtual Health Library (VHL), and Web of Science (WOS), following the PRISMA guidelines. Relevant observational studies were assessed for reporting using STROBE guidelines, whereas case reports were assessed using CARE guidelines. Independent reviewers evaluated studies based on eligibility criteria and analyzed relevant studies.

**Results:**

Postoperative complications observed in patients undergoing either technique (TMVR or TEER) included acute kidney injury, stroke, access site complications, and postoperative bleeding. These complications were generally more frequent in patients after TMVR. Myocardial infarction, prosthesis malposition, and valve migration were reported exclusively after TMVR, whereas ischemic cardiomyopathy was associated with TEER. Regarding hemodynamic outcomes, TMVR patients were associated with greater MR reduction to ≤ 1, frequently observed complete MR resolution, near‐laminar blood flow, and marked improvement in left ventricular function (assessed using LVEDVI and LVESVI). TMVR was associated with significantly higher 30‐day mortality, whereas pooled 1‐year mortality did not differ significantly between TMVR and TEER.

**Conclusion:**

Although TEER remains the safer option in appropriately selected patients, TMVR offers greater efficacy in MR resolution at the cost of higher procedural risk. However, findings should be interpreted with caution, given that patient selection between TMVR and TEER patients may influence observed outcomes.

## 1. Introduction

Mitral regurgitation affects 2% of the population globally, with a widely increasing prevalence, especially among the elderly [1]. In the United States alone, between 2 and 2.5 million people were affected in 2000, with this number estimated to double by 2030 [1]. Given the increasingly prevalent nature of MR, less invasive treatment approaches are being explored to reduce the morbidity and cost associated with open surgical techniques. The less invasive methods include both transcatheter edge‐to‐edge repair (TEER) and transcatheter mitral valve replacement (TMVR).

Although the most common treatment for mitral regurgitation is the surgical technique, there has been a recent shift toward less invasive and transcatheter approaches, including both TEER and TMVR. TEER is indicated in patients with suitable anatomy, yet a high surgical risk [2]. On the other hand, TMVR is recommended for patients with severe MR who are not candidates for TEER due to complex structural damage [2]. The annual number of mitral valve procedures performed between 2015 and 2020 in the United States has not changed [3]. However, the number of transcatheter mitral valve procedures performed in the United States has increased by 313%, whereas the number of surgical mitral valve treatments has decreased by 31.4% [4]. Surgical interventions that used to make up 91.8% of all mitral valve treatment options in the United States now comprise 65.0% of all therapies [3].

This systematic review is aimed at comparing indications, contraindications, techniques, intraoperative complications, postoperative complications, hemodynamic outcomes, long‐term clinical outcomes, and mortality in mitral valve regurgitation patients undergoing TEER and TMVR. As the transcatheter approach is becoming the future of mitral regurgitation therapies, more research needs to be conducted regarding the comparison between the two techniques. There is currently a lack of such data. Comparing these techniques is crucial for clinical decision‐making, hopefully making this review a reference for future studies.

## 2. Methods

### 2.1. Search Strategy

This systematic review was conducted in accordance with the Preferred Reporting Items for Systematic reviews and Meta‐Analyses (PRISMA) guidelines. Four databases were used to conduct the article search, which included PubMed, Cochrane Library, Virtual Health Library (VHL), and Web of Science (WOS). This search was completed on January 12, 2025, using the keywords “transcatheter mitral valve replacement (TMVR),” “transcatheter edge‐to‐edge repair (TEER),” and “mitral regurgitation (MR)” or all their applicable synonyms as well as MeSH terms if available (see Table S1).

The filtration process was conducted by three independent reviewers based on title and abstract, followed by a full‐text review based on eligibility criteria and the research question (comparing TMVR vs. TEER in MR patients). Additional papers were added to the review through website searching that met the eligibility criteria but did not appear in the original search of the four databases. Any disputes were resolved between the three reviewers, and if a dispute could not be settled, it was resolved by the senior author. No protocol was registered.

### 2.2. Eligibility Criteria

The inclusion criteria required that articles must first be written in English and be original research, such as randomized controlled trials, case reports, and observational studies, as well as in human populations. Lastly, the articles must compare both techniques, TMVR and TEER, in MR patients within the same study.

Articles excluded from this review were those written in languages other than English, nonoriginal research, such as literature reviews, systematic reviews, and meta‐analyses, and populations other than humans, such as those on animals, patients with tricuspid regurgitation, atrial septal defects, or mitral stenosis. Most importantly, articles that only studied one of the techniques, TMVR or TEER, without comparing the two methods, were removed.

### 2.3. Data Extraction

After articles were properly filtered, all relevant articles were uploaded to Zotero. From this list, the three independent reviewers extracted all demographic information, such as author and year, study design, population, age, gender, population characteristics, and type of mitral regurgitation. For each relevant article, the numbers of patients included in the TMVR and TEER groups were extracted separately.

The three reviewers also extracted data for the summary of outcomes, which included classification of MR, indications, technique, intraprocedure complications and incidence, postprocedure complications and incidence, risk factors, outcomes, and mortality. Intraprocedure complications included those arising during each procedure, and postprocedure complications included those related to the procedure arising after completion.

### 2.4. Quantitative Analysis

A quantitative analysis of the odds ratio of 30‐day mortality, 1‐year mortality, postoperative bleeding, and acute kidney injury between TEER and TMVR techniques was done using Cochrane RevMan, represented by various forest plots among the articles that reported such complications. Case reports were excluded from the quantitative analysis. These complications were selected because sufficient data were available to run a quantitative analysis despite heterogeneity among included articles, the small number of studies, and limited sample sizes. Other outcomes, such as stroke, access site complications, and MR reduction, could not be quantitatively synthesized because fewer than three studies reported comparable outcome definitions. Statistical significance was defined by a *p* value of less than 0.05. Heterogeneity was assessed using Cochrane *Q* and represented as the *I* ^2^ statistic. Given the clinical heterogeneity among included studies, all quantitative analyses were performed using random effects models.

### 2.5. Quality Assessment and Risk of Bias

Based on the study design, the articles were assessed using the EQUATOR guidelines. Observational studies reporting quality were assessed using the Strengthening the Reporting of Observational Studies in Epidemiology (STROBE) guidelines, and the case reports were assessed using the Case Report (CARE) guidelines. The risk of bias for observational studies was assessed using the Risk of Bias In Nonrandomized Studies of Interventions (ROBINS‐I), whereas the case reports were assessed using the JBI Critical Appraisal Checklist for Case Reports. These guidelines both ensure quality and standardization in medical research. They are aimed at producing clinically useful and rigorous reporting across all the different study types.

## 3. Results

### 3.1. Search Strategy Results

**Figure 1:**
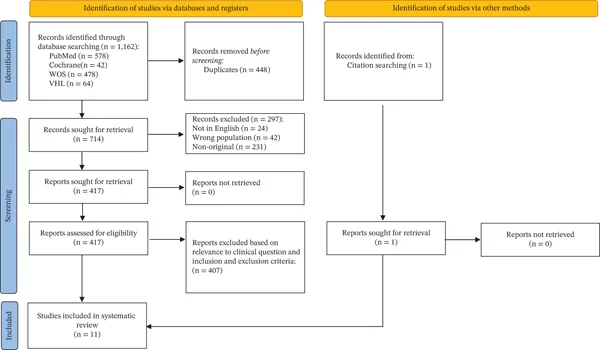
Flowchart following the PRISMA guideline.

An initial search of four electronic databases yielded a total of 1162 articles initially extracted for possible relevance in this systematic review. Following a careful screening process, 448 duplicates were removed, leaving 714 articles for further assessment. During the title and abstract review stage, 297 articles were excluded based on failure to meet the inclusion criteria. A subsequent full‐text review led to the exclusion of an additional 407 articles due to insufficient relevance to the clinical question, such as not directly comparing the two techniques in the same paper. A total of 10 articles were deemed suitable for inclusion, and 1 additional article was found through website searching, yielding a final total of 11 articles in this systematic review (Figure 1) [2, 4–13].

### 3.2. Demographics and Characteristics

There were 11 studies included in total; nine were observational studies, and two were case reports. The date range of the included studies spanned from 2022 [2, 4, 12] to 2024 [7, 9]. Across the 11 included studies, 4376 patients were included in the TMVR groups and 38,735 patients in the TEER groups. Of the TMVR patients in Mack et al. [12], 2551 underwent transseptal access, and 611 underwent transapical access. In Ben Ali et al. [4], 24 patients underwent transseptal access, and 205 underwent transapical access, whereas the remaining TMVR patients in the other studies underwent transapical access. All of the TEER patients underwent transseptal access. Mack et al. [12] had the largest population in both the TEER (33,878) and TMVR (3597) groups, whereas Scotti et al. [9] and Stolz et al. [8] had the smallest population in both (1). The mean age of the study population ranged from 66.7 to 84.7 years. Stolz et al. [8] had the highest mean age in both the TEER and TMVR groups (86), whereas Pugliese et al. [6] had the lowest mean age in both (69.5 ± 13 and 62.3 ± 16). Furthermore, the proportion of males was greater than that of women, with men making up 21,142 of the population. One study did not specify population characteristics [6]; three studies required mitral regurgitation Grade 3+ or higher [4, 7, 10], whereas the others required any form of mitral regurgitation [2, 5, 8, 9, 11–13]. Six of the studies addressed both types of mitral regurgitation [2, 5–7, 11, 13], four of the studies addressed only Type 2 [4, 8, 11, 12], and one study addressed only Type 1 [9]. Two of the studies were completely relevant [10, 11], whereas the others were partially relevant [2, 4–9, 12, 13] (Table 1).

**Table 1:** Demographics and characteristics.

Author, year	Study design	Number of population	Age	Gender	Population characteristics	Type of mitral regurgitation (1 = anatomical, 2 = functional)
Ben Ali et al., 2022 [4]	Retrospective study	TMVR: 229	TMVR: 78.5 (72.0–83.0)	TMVR: Male, 145; female, 84	MR ≥ 2+ that were not candidates for alternative therapies and passed TMVI evaluation	2
		TEER: 216	TEER: 78.5 (72.0–83.0)	TEER: Male, 124; female, 92		
Hungerford et al., 2022 [10]	Retrospective cohort study	TMVR: 46	TMVR: 72 ± 9	TMVR: Male, 34 (74%); female, 12 (26%)	MR Grade ≥ 3+ and LVEF ≤ 50%	Both 1 and 2
		TEER: 50	TEER: 80 ± 9	TEER: Male, 32 (64%); female, 18 (36%)		
Ludwig et al., 2022 [2]	Retrospective study	TMVR: 38	TMVR: 77.0 (72.9–80.1)	TMVR: Male, 22; female, 16	MR patient excluded pt. treated with transcatheter annuloplasty, MV surgery, and nonsevere MR	Both 1 and 2
		TEER: 28	TEER: 80.5 (76.4–84)	TEER: Male, 16; female, 12		
Ludwig et al., 2023 [11]	Retrospective study	TMVR: 235	TMVR: 75.5 (70–80)	TMVR: Male, 142; female, 93	TMVR if prior CABG and TEER if suitable anatomy	2
		TEER: 411	TEER: 76.7 (70.1–80.5)	TEER: Male, 243; female, 168		
Lurz et al., 2024 [7]	Prospective observational study	TMVR: 2	TMVR: 77.1 ± 9	TMVR: 2 (unspecified gender)	MR ≥ 3+, cleared by specialists as candidates for TEER using transthoracic/transesophageal echocardiography and clinical presentation	Both 1 and 2
		TEER: 528	TEER: 77.1 ± 9	TEER: Male, 321; female, 223		
Mack et al., 2022 [12]	Retrospective study	TMVR: 3597	TMVR: 75 (67–81)	TMVR: Male, 2164; female, 1433	Both TEER and TMVR were prohibited from surgical repair and replacement	2
		TEER: 33,878	TEER: 80.0 (73–85)	TEER: Male, 15,690; female, 18,186		
Pugliese et al., 2023 [6]	Prospective observational study	TMVR: 3	TMVR: 62.3 ± 16	TMVR: Male, 1; female, 2	—	Both 1 and 2
		TEER: 2	TEER: 69.5 ± 13	TEER: Male, 2; female, 0		
Scotti et al., 2024 [9]	Case report	TMVR: 1	TMVR: 81	TMVR: Male, ‐; female, unspecified	Prior TEER needs to be fixed with TMVR: Severe symptomatic MR and circumferential mitral annular calcification	1
		TEER: 1	TEER: 81	TEER: Male, ‐; female, unspecified		
Stolz et al., 2023 [8]	Case report	TMVR: 1	TMVR: 86	TMVR: Female	Severe secondary MR	2
		TEER: 1	TEER: 86	TEER: Female		
Sudo et al., 2023 [5]	Retrospective cohort study	TMVR: 14	TMVR: 77.7 ± 8.3	TMVR: Male, 131; female, 143	Excluded patients requiring revascularization for coronary artery disease, cardiac resynchronization therapy, or interventions for other valve diseases before mitral valve interventions	Both 1 and 2
		TEER: 250	TEER: 77.7 ± 8.3	TEER: Male, 131; female, 143		
Wang et al., 2023 [13]	Retrospective cohort study	TMVR: 210	TMVR: 69.6 ± 13.09	TMVR: Male, 108 (51.4%); female, 102 (48.6%)	MR	Both 1 and 2
		TEER: 3370	TEER: 75.99 ± 11.78	TEER: Male, 1836 (54.5%); female, 1534 (45.5%)		

Abbreviations: CABG, coronary artery bypass surgery; MR, mitral regurgitation; TEER, transcatheter edge‐to‐edge repair; TMVR, transcatheter mitral valve.

### 3.3. Summary of Outcomes

The classification of mitral regurgitation was 3+ or greater in 10 studies [2, 4–12] and 1+ in one [13]. The indications for the use of TMVR were unspecified in three [5, 12, 13], severe symptomatic MR in seven [2, 4, 6–8, 10, 11], and fibrocalcific leaflet change in one [9]. The indications for TEER were unspecified in two [5, 13] and indicated by suitable anatomy in nine [2, 4, 6–12]. The technique used for TMVR was the Tendyne prosthesis in seven studies [2, 5–8, 10, 13], Laceration of the Anterior Mitral Leaflet to Prevent Outflow Obstruction (LAMPOON) in one [12], 10 different unspecified devices in one [4], percutaneous transseptal using Cephea in one [9], and unspecified in one [11]. The technique used for TEER was MitraClip in all studies [2, 4–13]. Intraprocedure complications included 22 different complications, the most notable including leaflet tear, severe bleeding, and electrolyte disorders. For instance, in Hungerford et al. [10], TEER had no reported intraprocedure complications, whereas TMVR reported 2.2% of patients experiencing hemodynamic compromise. Mack et al. [12] also reported major bleeding in 7.8% of patients undergoing TEER, whereas TMVR patients experienced minor complications like hemolysis and paravalvular leak at 2.2% each (Table 2).

**Table 2:** Summary of outcomes.

Author, year	Classification for MR (mild, moderate, severe)	Indications (TEER vs. TMVR)	Technique	Intraprocedural complications and incidence	Postprocedure complications and incidence	Risk factors (age, obesity, diabetes)	Outcomes	Mortality
Ben Ali et al., 2022 [4]	Moderate–severe (≥ 2+)	TMVR: Symptomatic MR ≥ 2+ that were unsuitable for other therapies	TMVR: 10 different TMVI devices	TMVR: Access site complications, 21 patients (9.6%); bleeding reintervention, 12 (7.5%)	TMVR: Reintervention for bleeding, 12 patients (7.5%); acute renal failure, 30 patients (15.4%); disabling stroke, 6 patients (3.0%); access site complications, 18 patients (9.6%); LVOT obstruction, 6 patients (3.4%); prosthesis malposition, 6 patients (3.4%); valve migration, 3 patients (1.5%)	—	TMVR: 95.1% of patients achieved an MR severity of ≤ 1+. Full MR elimination was present in 83.9% of patients	30 days: TMVR: CV mortality, 19 patients (8.7%); all‐cause mortality, 22 patients (9%)
		TEER: Severe primary MR deemed at high surgical risk and secondary MR on medical therapy	TEER: MitraClip or PASCAL system	TEER: Access site complications, 7 patients (3.4%); bleeding reintervention, 1 (0.7%)	TEER: Reintervention for bleeding, 1 patient (0.7%); acute renal failure, 5 patients (2.6%); disabling stroke, 3 patients (1.5%); access site complications, 6 patients (3.4%)		TEER: 52.6% MR elimination, residual MR was less than ≤ 1+ in all patients	TEER: CV mortality, 6 patients (3%); all‐cause mortality, 9 patients (4.3%)
Hungerford et al., 2022 [10]	Severe (≥ 3+)	TMVR if severe symptomatic MR	TMVR: Tendyne prosthesis	—	—	—	TMVR: MR was eliminated in all patients	TMVR: 9 patients
		TEER if suitable anatomy	TEER: MitraClip				TEER: MR ≤ 2+ in 92% of patients at discharge	TEER: 5 patients
Ludwig et al., 2022 [2]	Severe (≥ 3+)	TMVR if prior CABG	TMVR: Transapical (Tiara and Tendyne valves); transeptal (CardiAQ, HighLife)	TMVR: Atrial migration requiring surgery, 1 patient; temporary left atrial tamponade, 1 patient; acute‐on‐chronic kidney failure, 1 patient	TMVR: MI, 1 patient; renal failure, 3 patients; major access site complications, 9 patients; major bleeding, 7 patients	Smoking history: TMVR, 19; TEER, 12	TMVR: MR was reduced to mild (24.2%) or none/trace MR (75.8%) in all patients	30 days: TMVR, 4 patients (1 procedural death and 3 in‐hospital deaths); TEER: ‐ 1 year: TMVR, 13 patients; TEER, 8 patients
						Arterial HTN: TMVR, 19;		
							TEER: MR was reduced to mild (55.6%) or none/trace (18.5%) in the majority of patients at discharge, whereas in 18.5% of these patients, MR was still moderate, and 7.4% remained with severe MR	
						TEER, 20		
						Diabetes: TMVR, 8;		
		TEER if suitable anatomy	TEER: Bailout TEER	TEER: ‐	TEER: ‐	TEER, 6		
						Coronary artery disease: TMVR, 22; TEER, 16		
Ludwig et al., 2023 [11]	Severe (≥ 3+)	TMVR if prior CABG	TMVR: ‐	—	—	HTN: TMVR, 167; TEER, 281	1‐year mortality rates were higher after TMVR, but it was a more complete and durable way to treat MR	Procedural mortality: TMVR, 4 patients (1.7%); TEER, 2 patients (0.5%)
		TEER if suitable anatomy	TEER: MitraClip			Diabetes: TMVR, 69; TEER, 110	TEER was a better outcome for patients over 75, female patients, patients with LVEF > 30%, patients without RV dysfunction, and without TR	30 days: TMVR, 16 patients (6.8%); TEER, 16 patients (3.8%)
								1 year: TMVR, 61 patients (25.8%); TEER, 78 patients (18.9%)
Lurz et al., 2024 [7]	Mild, moderate, and severe	TMVR: Unsuccessful TEER due to high gradient after leaflet grasping	TMVR: Tendyne valve	TMVR: ‐	TMVR: ‐	—	TMVR: MR reduction was observed and sustained at 1 year postoperatively, with 98% of patients attaining MR ≤ 2+	TMVR: ‐
		TEER: Symptomatic, degenerative, or functional MR who are at high risk of surgery	TEER: MiCLASP, MitraClip (2)	TEER: Access site and vascular complications, 6 patients (1.1%)	TEER: Stroke 0.9%, MI 0.2%, mitral valve reintervention 0.4%, renal complications 1.1%, bleeding 4%, device embolization 0.2%		82.6% MR ≤ 1+. Survival was 87.3% using Kaplan–Meier estimates, and the absence of HF hospitalization admission was 84.3%	TEER: 30‐day CV mortality, 6 patients (1.1%); 1‐year CV mortality, 45 patients (8.3%)
							TEER: ‐	
Mack et al., 2022 [12]	Moderate to severe (3+)	TMVR: ‐	TMVR: LAMPOON procedure	TMVR: Major bleeding that required blood transfusion in 7.8%	TMVR: Device embolization 0.8%, device thrombosis 0.4%, other device‐related event 0.9%, MI 0.5%, TIA 0.4%, stroke 1.9%	Diabetes (34% of pts)	TMVR: 99.4% none or mild MR after 30 days and 0.6% moderate–severe/severe	30 days: TMVR, 223 patients (6.2%); TEER, 1389 patients (4.1%)
		TEER: Frailty, hostile chest, severe liver disease, porcelain aorta, STS Risk 6, STS Risk 8, unusual extenuating circumstance, right ventricular dysfunction with severe tricuspid valve regurgitation, chemotherapy for malignancy, major bleeding diathesis, immobility, AIDS, severe dementia, high risk of aspiration, and the internal mammary artery at high risk for injury	TEER: MitraClip	TEER: Major bleeding that required blood transfusion in 7.8%	TEER: Single leaflet attachment 1.3%, device embolization 0.1%, device thrombosis 0%, other device‐related event 0.6%, MI 0.2%, TIA 0.3%, stroke 1.3%	Obesity: BMI > 30 (29% of pts)	TEER: 91.3% none or mild MR after 30 days and 8.7% moderate–severe/severe	1 year: TMVR, 809 patients (22.5%); TEER, 7826 patients (23.1%)
Pugliese et al., 2023 [6]	Severe (≥ 3+)	TMVR: Functional MR with severe cardiomyopathy	TMVR: Tendyne prosthesis	—	TMVR: None	HFrEF	TMVR: Near physiological flow patterns	—
		TEER: Functional MR with ischemic cardiomyopathy	TEER: MitraClip		TEER: Both patients had ischemic cardiomyopathy		TEER: Mild‐to‐moderate MR after procedure and turbulent flow patterns	
Scotti et al., 2024 [9]	Severe (≥ 3+)	TMVR: Fibrocalcific leaflet changes and loss of lateral edge‐to‐edge sutures	TMVR: Percutaneous transeptal using Cephea transseptal delivery system to implant 36 mm valve	TMVR: ‐	—	Age and mitral annular calcification	—	—
		TEER: ‐	TEER: ‐	TEER: Single leaflet device attachment, leaflet perforation, and no implantation due to MR reduction				
Stolz et al., 2023 [8]	Severe (3+)	TMVR: Chordal rupture next to the clip device	TMVR: Tendyne valve	—	—	—	—	—
		TEER: Severe secondary MR	TEER: MitraClip, ELASTA‐Clip					
Sudo et al., 2023 [5]	Moderate to severe (3+) and severe (≥ 3+)	—	TMVR: Tendyne, HighLife, and SAPIEN 3	TMVR: ‐	—	—	TMVR: MR ≤ 1+ at discharge, 10 (71.4%)	1 year: TMVR and TEER, 20.2%
			TEER: MitraClip and PASCAL	TEER: 32 pt/has device implantation failure			TEER: MR ≤ 2+ at discharge, 213 (86.4%)	
Wang et al., 2023 [13]	Mild (1+)	—	TEER: MitraClip	—	TMVR: Blood transfusion (16.2% vs. 5.0%), electrolyte disorders (31.9% vs. 11.3%), cardiogenic shock (11.4% vs. 3.7%), cardiac arrest (2.9% vs. 1.0%), respiratory failure (3.8% vs. 0.9%), respiratory complications (3.8% vs. 1.7%), mechanical ventilation use (10.5% vs. 3.7%), thrombosis due to cardiac prosthetic devices (1.4% vs. 0.2%), acute kidney injury (22.9% vs. 13.3%)	—	—	In‐hospital mortality: TMVR, 17 patients (8.1%); TEER, 64 patients (1.9%)

Abbreviations: TEER, transcatheter edge‐to‐edge repair; TMVR, transcatheter mitral valve repair.

Postprocedural complications included 22 different complications, the most notable including leaflet tear, severe bleeding, and electrolyte disorders. Ben Ali et al. [4] found that 15.4% of TMVR patients experienced acute renal failure and 3.0% suffered disabling strokes, whereas TEER had a lower incidence of these complications. Lurz et al. [7] noted that TMVR patients had a 4% incidence of bleeding complications, whereas TEER patients experienced vascular complications in 1.1% of cases.

### 3.4. Quantitative Analysis Results

**Figure 2:**
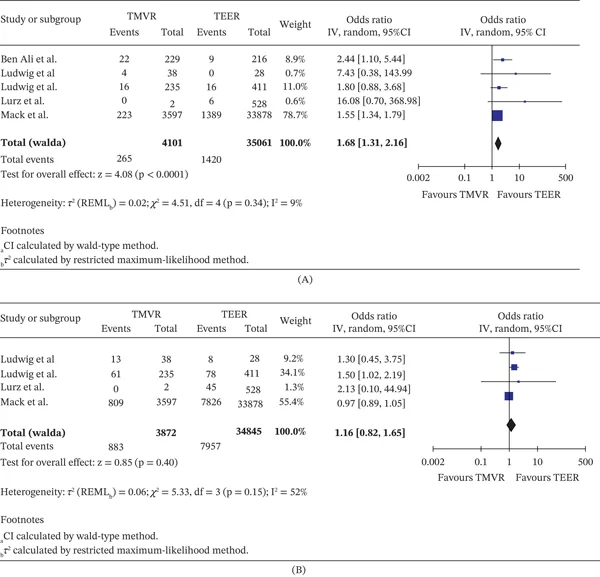
(A) Forest plot showing quantitative analysis of 30‐day mortality between TMVR and TEER. (B) Forest plot showing quantitative analysis of 1‐year mortality between TMVR and TEER.

**Figure 3:**
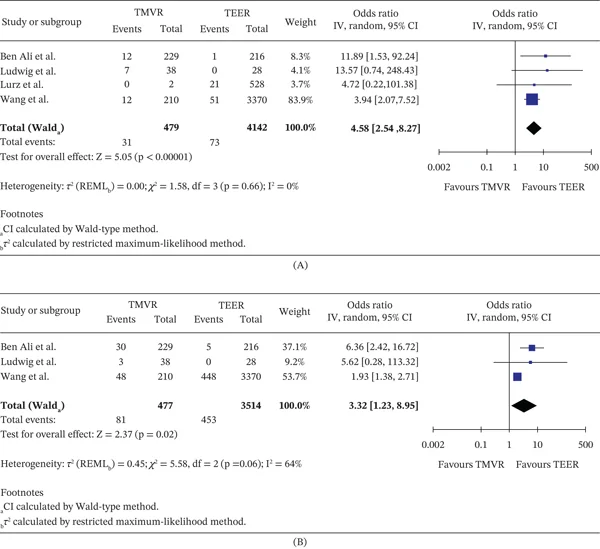
(A) Forest plot showing quantitative analysis of postoperative bleeding between TMVR and TEER. (B) Forest plot showing quantitative analysis of AKI between TMVR and TEER.

Quantitative analysis demonstrated notable differences in both mortality and complication rates between TMVR and TEER techniques. Patients undergoing TMVR had a significantly higher risk of 30‐day mortality, with a 68% increase in the odds compared with those undergoing TEER (OR 1.68, 95% CI [1.31, 2.16], *p* < 0.0001) (Figure 2A) [2, 4, 7, 11, 12]. There was no statistically significant difference in 1‐year mortality between TMVR and TEER (OR 1.16, 95% CI [0.82, 1.65], *p* = 0.40) (Figure 2B) [2, 7, 11, 12]. In terms of postoperative complications, TMVR was associated with significantly greater odds of postoperative bleeding, which occurred 4.58 times more frequently when compared with TEER patients (OR 4.58, 95% CI [2.54, 8.27], *p* < 0.00001) (Figure 3A) [2, 4, 7, 13]. Additionally, acute kidney injury following surgery was found to be more common among TMVR patients, with 3.32 times the odds in comparison to TEER patients (OR 3.32 95% CI [1.23, 8.95],*p* = 0.02) (Figure 3B) [2, 4, 13].

### 3.5. Quality Assessment and Risk of Bias Results

The reporting quality assessment of the studies included in this systematic review was conducted following the appropriate guidelines based on the study design. All nine observational studies were assessed using the STROBE guideline (Table 3) and ROBINS‐I (Table 4) [2, 4–7, 10–13]. The two case reports were assessed using the CARE guidelines (Table S2) and JBI Critical Appraisal Checklist (Table S3) [8, 9].

**Table 3:** TROBE guidelines.

Author, year	1a	1b	2	3	4	5	6a	6b	7	8	9	10	11	12a	12b	12c	12d	12e	13a	13b	13c	14a	14b	14c	15	16a	16b	16c	17	18	19	20	21	22
Ben Ali et al., 2022 [4]	Y	Y	Y	Y	Y	Y	Y	—	Y	Y	Y	Y	Y	Y	Y	N	N	N	Y	Y	Y	Y	N	Y	Y	Y	Y	—	Y	Y	Y	Y	N	Y
Hungerford et al., 2022 [10]	—	—	Y	Y	Y	Y	Y	N	Y	N	Y	Y	Y	Y	—	Y	Y	N	N	N	Y	Y	—	Y	Y	Y	—	—	Y	Y	N	Y	N	Y
Ludwig et al., 2022 [2]	N	Y	Y	Y	Y	Y	Y	—	Y	Y	N	N	Y	Y	Y	N	N	N	Y	N	Y	Y	—	Y	Y	Y	Y	—	Y	Y	Y	Y	N	N
Ludwig et al., 2023 [11]	Y	Y	Y	Y	Y	Y	Y	Y	Y	Y	Y	N	Y	Y	Y	Y	Y	Y	Y	N	Y	Y	N	Y	Y	Y	Y	—	Y	Y	Y	Y	N	Y
Lurz et al., 2024 [7]	Y	Y	Y	Y	Y	N	Y	—	Y	Y	N	N	Y	Y	N	Y	N	N	Y	Y	Y	Y	N	Y	Y	Y	Y	—	Y	Y	Y	Y	N	Y
Mack et al., 2022 [12]	N	Y	Y	Y	Y	Y	Y	—	Y	N	Y	Y	—	N	N	Y	Y	N	Y	N	N	Y	N	Y	Y	Y	N	—	N	Y	Y	Y	N	Y
Pugliese et al., 2023 [6]	N	Y	Y	Y	Y	Y	Y	—	Y	Y	N	N	—	N	N	N	N	N	N	—	N	Y	—	N	Y	N	N	—	N	Y	Y	Y	N	N
Sudo et al., 2023 [5]	N	Y	Y	Y	Y	Y	Y	—	Y	Y	N	N	Y	Y	N	N	N	Y	Y	Y	Y	Y	N	N	Y	Y	Y	—	Y	Y	Y	Y	N	Y
Wang et al., 2023 [13]	N	Y	Y	Y	Y	Y	Y	—	N	N	N	N	Y	Y	N	N	N	N	N	N	Y	Y	N	N	Y	Y	N	—	Y	Y	Y	Y	N	Y

*Note:* The quality assessment was conducted using the STROBE (Strengthening the Reporting of Observational Studies in Epidemiology) checklist. The items represent the following: 1a, identify the study′s design within the title or abstract; 1b, present an informative and balanced abstract; 2, describe the scientific basis and rationale of the study; 3, specify the study′s objectives and hypotheses; 4, detail key aspects of the study design; 5, detail the location(s), setting, and dates relevant to the study; 6a, outline the inclusion/exclusion criteria as well as methods used to select participants; 6b, for matched studies, outline what criteria were used for matching participants; 7, define outcomes, exposures, predictors, and confounders; 8, detail the source(s) of data collection and the method(s) by which they are measured; 9, identify possible biases that could be present; 10, detail how the sample size for the study was determined; 11, detail how quantitative variables were handled; 12a, detail the statistical methodology utilized, with particular emphasis on confounder adjustment; 12b, detail subgroup and interaction analysis methodologies; 12c, detail methods of dealing with missing data; 12d, detail methods of addressing loss‐to‐follow‐up/matching issues; 12e, detail methodologies employed in conducting sensitivity analyses; 13a, report number of participants at each study phase; 13b, provide explanation for nonparticipants; 13c, consider utilizing a flow diagram; 14a, detail participant demographics as well as exposure status; 14b, identify missing data; 14c, summarize cohort study follow‐up time; 15, detail the findings regarding the outcome/exposure variable(s); 16a, report both unadjusted and adjusted estimates; 16b, report category bounds for continuous variables; 16c, translate relative risk to absolute risk when applicable; 17, detail additional analytical approaches such as subgroup and interaction analysis; 18, summarize key findings; 19, discuss study limitations; 20, offer cautious interpretations of findings; 21, discuss applicability to other populations; 22, list funding sources and role of sponsors. The symbol “—” indicates unclear/unavailable.

Abbreviations: CC, case control; CS, cross‐sectional; N, no; PC, prospective cohort; RC, retrospective cohort; Y, yes.

**Table 4:** ROBINS‐I.

Author, year	D1	D2	D3	D4	D5	D6	Overall
Ben Ali et al., 2022 [4]	Serious	Low	Serious	Moderate	Moderate	Moderate	Serious
Hungerford et al., 2022 [10]	Serious	Low	Moderate	Moderate	Moderate	Moderate	Serious
Ludwig et al., 2022 [2]	Serious	Low	Moderate	Serious	Moderate	Moderate	Serious
Ludwig et al., 2023 [11]	Serious	Low	Moderate	Moderate	Moderate	Low	Serious
Lurz et al., 2024 [7]	Serious	Low	Moderate	Moderate	Low	Moderate	Serious
Mack et al., 2022 [12]	Serious	Low	Moderate	Low	Moderate	Moderate	Serious
Pugliese et al., 2023 [6]	Serious	Low	Moderate	Low	Moderate	Moderate	Serious
Sudo et al., 2023 [5]	Serious	Low	Moderate	Moderate	Moderate	Moderate	Serious
Wang et al., 2023 [13]	Serious	Low	Moderate	Moderate	Low	Moderate	Serious

*Note:* Domains: D1, bias due to confounding; D2, bias in classification of interventions; D3, bias in selection of participants into the study; D4, bias due to missing data; D5, bias in measurement of the outcome; D6, bias in selection of the reported result. Judgment: Low, low risk of bias; Moderate, moderate risk of bias; Serious, serious risk of bias; Critical, critical risk of bias.

Using the ROBINS‐I tool to evaluate the risk of bias in the included observational studies, all of the included articles were found to have a serious risk of bias due to confounding variables and a low risk for classification of interventions [2, 4–7, 10–13]. All articles were found to have a moderate risk of bias for the participant selection process, except for Ben Ali et al., which had a serious risk of bias [4]. Most articles were found to have a moderate risk of bias for missing data, except for Ludwig et al., which had a serious risk of bias, and both Mack et al. and Pugliese et al., which both had a low risk of bias [2, 6, 12]. Assessing risk of bias in measurement of the outcome demonstrated that all articles had a moderate risk of bias except for Lurz et al. and Wang et al., which had a low risk of bias [7, 13]. Ludwig et al. demonstrated a low risk of bias in the selection of the reported result, whereas all other articles showed a moderate risk [11]. Overall, all included observational studies were found to have a serious risk of bias overall [2, 4–7, 10–13].

According to the JBI Critical Appraisal Checklist for Case Reports, both articles displayed an overall low risk of bias [8, 9]. The patient′s demographic characteristics, history, clinical presentation, diagnostic assessment, interventions, postintervention outcomes, and key takeaway lessons were all clearly demonstrated in both case reports [8, 9]. However, the identification of adverse or anticipated events was not described in either case report [8, 9]. Regardless, because the remainder of the JBI criteria was fulfilled, the overall risk of bias remained low for both case reports [8, 9].

## 4. Discussion

### 4.1. Indications and Contraindications

**Figure 4:**
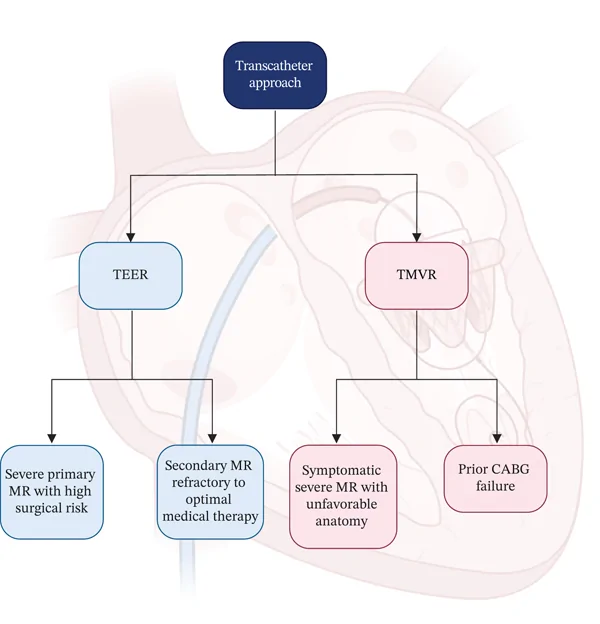
Algorithm for selecting a transcatheter technique for MR patients. Created in BioRender (Basson, M. [2025]; https://BioRender.com/u42q8eg).

The main indication for TEER is severe primary MR patients considered to be at high surgical risk, as well as those with secondary MR that do not respond to optimal medical therapy [14]. The method requires a favorable mitral valve anatomy, which means there must be suitable leaflet morphology and the absence of prohibitive leaflet calcification [15]. TEER is frequently chosen for patients with frailty, adverse chest conditions, severe liver disease, porcelain aorta, or high surgical risk scores [15]. Additionally, in clinical situations like right ventricular dysfunction accompanied by severe tricuspid regurgitation, history of chemotherapy, significant bleeding predisposition, lack of movement, and various other comorbid conditions, TEER is preferred because it is less invasive and can cater to complex patient profiles [15] (Figure 4).

Primary MR arises from intrinsic structural abnormalities of the mitral valve, such as leaflet prolapse, flail, or chordal rupture. TEER is particularly suited for these patients when surgical risk is high and leaflet anatomy is favorable. Functional MR, in contrast, results from geometric changes in the heart rather than intrinsic valve disease. Atrial functional MR (AFMR) is driven by left atrial enlargement with relatively preserved left ventricular function, whereas ventricular functional MR (VFMR) occurs due to left ventricular dilation and systolic dysfunction. TEER is often appropriate for AFMR and some VFMR cases when leaflet anatomy allows effective grasping of the leaflets to reduce regurgitation [16].

Conversely, TMVR is recommended for symptomatic patients with severe MR who are not suitable candidates for TEER, often because of unfavorable mitral valve anatomy or previous procedural failure [17]. Particular anatomical characteristics that direct TMVR selection include fibrocalcific alterations of leaflets, chordal rupture near previous clip devices, or the loss of lateral edge‐to‐edge sutures after TEER [18]. After a failed TEER, TMVR can act as a salvage therapy, especially in situations where there are significant residual transmitral gradients following leaflet grasping [11]. TMVR is also preferred for patients who have undergone previous cardiac surgeries, including coronary artery bypass grafting and valve replacement, due to the increased surgical risks linked to repeat sternotomy and hostile chest anatomy [18]. TMVR is more frequently utilized in patients with severe cardiomyopathy and extensive leaflet pathology, whereas TEER is often selected for functional MR secondary to ischemic cardiomyopathy with suitable leaflet anatomy [18]. In these patients, especially with VFMR or complex AFMR, TMVR may be favored because TEER may not provide sufficient reduction of MR [16].

In terms of procedural access, TMVR has historically been performed predominantly via a transapical approach, though transseptal approaches have become increasingly common with newer devices [11]. This distinction is clinically relevant, as transseptal TMVR offers a less invasive alternative that may reduce procedural morbidity and recovery time, whereas transapical TMVR allows more direct access to the mitral annulus, which may be advantageous in complex anatomical scenarios [11].

### 4.2. Technique

#### 4.2.1. TEER

The procedural methods for TEER and TMVR vary significantly due to the unique technical challenges and device designs associated with each procedure. Of the included studies reviewed, TEER was mainly carried out with the MitraClip system, whereas the PASCAL and ELASTA‐Clip devices were used occasionally. The MitraClip, recognized as the most used TEER device, utilizes a transseptal approach through femoral venous access to grasp and bring together the anterior and posterior mitral leaflets at the location of regurgitation, usually focusing on the A2–P2 scallops [19]. This edge‐to‐edge repair replicates the surgical Alfieri stitch and mitigates mitral regurgitation by improving leaflet coaptation [19]. The PASCAL system similarly employs a transseptal technique but offers a distinct design with a central spacer and wider paddles to optimize leaflet capture and reduce leaflet stress, which may confer advantages in select anatomies [19].

#### 4.2.2. TMVR

TMVR techniques show more heterogeneity, mirroring the variety of valve prostheses and delivery systems that are available. Various studies utilized devices like the Tendyne prosthesis, HighLife, CardiAQ, Tiara, Cephea, and SAPIEN 3 valves [20]. It is worth noting that the SAPIEN M3 valve was the first TMVR system to provide femoral venous access [21]. These devices were delivered via different access routes, including transapical and transseptal approaches [20]. The transapical approach, which involves directly puncturing the ventricle through the chest wall, was mainly employed for devices such as Tendyne and Tiara valves [20]. This method provides direct and stable access to the mitral annulus but necessitates a minithoracotomy [20]. In contrast, the CardiAQ, Cephea, and HighLife transseptal TMVR devices represent a less invasive approach, utilizing percutaneous delivery through femoral venous access with transseptal puncture [20]. Historically, HighLife was a transapical technique and is now transseptal [16]. This method relates to TEER access and has the potential to decrease procedural morbidity [20].

To reduce the risk of left ventricular outflow tract (LVOT) obstruction, a recognized limitation of TMVR resulting from anterior leaflet displacement, some TMVR cases included complex techniques like the LAMPOON procedure [20]. This underscores the procedural complexities that are distinct to TMVR in contrast to TEER. Along with variations in devices and access, certain studies noted bailout TEER or repeat TEER procedures as rescue strategies, highlighting the adaptability of TEER in procedural planning.

### 4.3. Operative Complications

#### 4.3.1. Prosthesis Malposition

Prosthesis malposition occurred in TMVR cases, highlighting the technical challenges involved in securely anchoring a prosthetic valve within the mitral annulus [5]. In contrast to the aortic position, the mitral annulus is a structure that is highly irregular, noncircular, and often extensively calcified, which complicates device seating [20]. Inadequate valve function, residual mitral regurgitation, or paravalvular leaks may result from malposition, jeopardizing the clinical benefit of TMVR [20]. These difficulties could stem from problems related to the size of the valve, how accurately it is deployed, or how the device is handled during the procedure [20]. Malposition is typically categorized as Clavien–Dindo Grade IIIb or IV. Access type may also influence the risk of malposition. Transapical TMVR provides direct access to the mitral annulus, which can facilitate more precise device deployment in patients with challenging anatomy but carries procedural risks related to thoracotomy and apical puncture [11]. Transseptal TMVR, increasingly used in contemporary practice, offers a less invasive route that may reduce perioperative morbidity, but the indirect approach can increase technical complexity in valve positioning [18]. It is necessary to improve device designs through accurate sizing and using techniques like preprocedural imaging and intraoperative verification with TEE fluoroscopy [20].

#### 4.3.2. Access Site Complications

Postoperative complications at the access site occurred in between 9.6% and 23.7% of patients after TMVR [3, 5], but in only 1.1%–3.4% of TEER patients [5, 8]. This highlights the persistence of intraprocedural vascular trauma and underscores the critical need for vascular closure, especially in TMVR. The higher incidence of access‐related complications in TMVR is likely due to the complexity of the procedure and the more invasive nature of the access routes used. TMVR is often performed transapically, which necessitates surgical entry through the chest wall and direct puncture of the left ventricular apex [20]. This provides direct access to the valve, but it carries inherent dangers, including myocardial injury, pericardial bleeding, apical hematoma, and complications related to the wound [20]. The transseptal access may reduce the risk of surgical wound complications and promote faster recovery, but it introduces technical challenges due to indirect catheter navigation and reliance on imaging guidance for precise valve deployment [11].

TEER, in contrast, is mainly conducted via transfemoral venous access using catheters and devices with smaller profiles, which reduces vascular disruption and procedural trauma [20]. Whereas transseptal puncture is utilized in TEER as well, the reduced device size and less invasive characteristics of the technique lead to a lower incidence of access site complications [20]. Additionally, venous access is typically more forgiving than arterial or transapical routes, presenting a lower risk of complications such as pseudoaneurysm, arteriovenous fistula, and major bleeding [20].

Complications at the access site for both procedures can manifest as hematoma, bleeding that necessitates transfusion, pseudoaneurysm development, or retroperitoneal hemorrhage [20]. This is especially true when there are errors or complications in the application of femoral artery access [20]. In TMVR patients, surgical wound dehiscence or apical bleeding may require reoperation or pericardial drainage, which increases morbidity [20].

**Figure 5:**
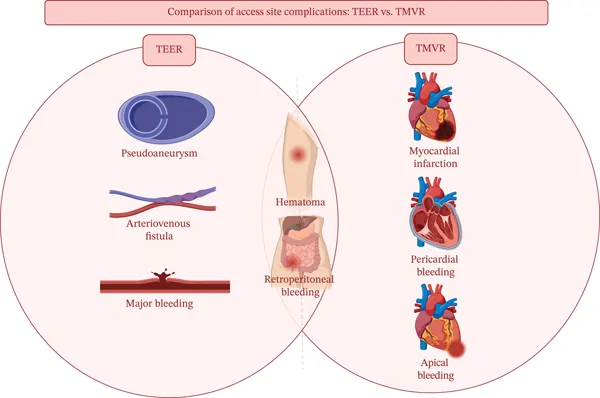
Comparison of access site routes and complications: TEER versus TMVR. Created in BioRender (Basson, M. [2025]; https://BioRender.com/dd75hgd).

In addition to increasing resource use and lengthening hospitalization, these complications have prognostic implications for older patients with limited physiologic reserve [20]. These complications are classified as Clavien–Dindo Grade III or IV depending on severity. The results highlight the necessity of ultrasound‐guided access, smaller sheath sizes, and meticulous closure techniques to mitigate such complications [20] (Figure 5).

### 4.4. Postoperative Complications

#### 4.4.1. Acute Kidney Injury

Acute kidney injury following surgery was a notable complication that occurred more commonly in TMVR than TEER. This disparity likely reflects several interconnected factors. TMVR procedures typically require larger amounts of iodinated contrast for planning the procedure, imaging during the procedure, and evaluating after implantation [22]. TEER, on the other hand, typically uses transesophageal echocardiography for guidance and features minimal or no use of contrast, which reduces this risk [22].

TEER typically has a shorter duration and is hemodynamically better tolerated, which may help explain the lower incidence of postoperative renal dysfunction observed. Mitigation of contrast use, optimization of hydration, monitoring of renal function, and avoidance of nephrotoxic agents are therefore all important strategies to minimize this adverse effect [22] (Table 5).

**Table 5:** Postoperative complications.

Complication	Clavien–Dindo classification	Mitigation strategy
Acute renal failure	Grade II or IV	Minimize contrast use, optimize hydration, monitor renal function pre/postop, and avoid nephrotoxic agents [22]
Stroke	Grade II or IV	Anticoagulation, cerebral embolic protection devices, meticulous catheter manipulation [23]
Access site complications	Grade IIIb or IV	Ultrasound‐guided access, smaller sheath sizes, meticulous closure techniques [20, 24]
Bleeding	Grade II	Careful anticoagulation management, hemostatic closure, and transfusion protocols [24]
Myocardial infarction	Grade II	Postprocedural ECG monitoring, preprocedure coronary assessment, and management of anticoagulation [25]
Renal failure	Grade IIIb	Same as acute renal failure [22]
Valve migration	Grade IV	Careful device positioning, confirm placement with imaging before release [20, 24]
Prosthesis malposition	Grade IV	Accurate sizing, preprocedural imaging, intraoperative verification with TEE/fluoroscopy [20, 24]
LVOT	Grade IV	Preop CT planning, consider LAMPOON, septal ablation when indicated [26]

*Note:* Clavien–Dindo classification of surgical complications. Grade I: Interventions vary from standard postoperative instructions with no radiological, surgical, and endoscopic interventions, or pharmacological treatment. Electrolytes, physiotherapy, antiemetics, analgesics, antipyretics, and diuretics may be used. CD classification: Grade II, drugs other than those used for Grade I complications, total parenteral nutrition, and blood transfusions; Grade III, radiological, endoscopic, or surgical intervention needed; Grade IIIa, no general anesthesia; Grade IIIb, general anesthesia; Grade IV, ICU admission with potentially fatal complications; Grade IVa, affecting a single organ; Grade IVb, affecting multiple organ; Grade V, mortality.

Abbreviations: ECG, electrocardiogram; TEE, transesophageal echocardiogram.

#### 4.4.2. Stroke

Strokes were more common after TMVR than TEER, aligning with the greater invasiveness and technical demands of TMVR. This increased risk may reflect both anatomical and procedural factors. In patients with mitral annular calcification (MAC), which can act as a source of embolic material, TMVR usually entails the manipulation of the heavily calcified mitral annulus [23].

Strokes are classified as Clavien–Dindo Grade III or IV depending on severity and neurologic impact. Postoperative stroke significantly affects structural heart procedures, frequently resulting in prolonged hospitalization, reduced quality of life, and heightened long‐term mortality rates [23]. Even minor strokes or transient ischemic attacks (TIAs) can hamper postoperative recovery and rehabilitation, especially in elderly patients who make up the majority of those undergoing mitral valve interventions [23]. Considering these findings, it is important to prioritize stroke risk stratification and mitigation in procedural planning, particularly for TMVR candidates. Embolic risk may be reduced by methods like anticoagulation, cerebral embolic protection devices, and meticulous catheter manipulation [23].

#### 4.4.3. LVOT

TMVR was linked to various valve‐related complications, particularly LVOT obstruction, which manifested in 3.4% of cases [5]. Obstruction of the LVOT is a serious complication arising solely from TMVR; it can threaten life and stems from the anterior mitral leaflet or a large prosthetic valve frame being displaced into the outflow tract. In patients with small left ventricular cavities, septal hypertrophy, or significant MAC, the anatomical relationship between the mitral valve and LVOT is particularly intricate, as these conditions can further constrict the LVOT during valve deployment [26]. This obstruction can hemodynamically resemble the dynamic left ventricular outflow gradients observed in hypertrophic cardiomyopathy, resulting in hypotension, low cardiac output, or even cardiogenic shock [26]. The risk of LVOT obstruction may vary between access routes. Transapical TMVR provides more direct control over valve orientation and deployment but involves larger manipulation within the left ventricle (LV), which could increase the potential for anterior leaflet displacement into the LVOT [11]. Transseptal TMVR, although less invasive and performed via femoral venous access, still carries the risk of LVOT obstruction because the prosthetic valve frame may extend into the outflow tract during deployment, particularly in patients with small ventricles or prominent septal hypertrophy [18]. LVOT obstruction is classified as a Clavien–Dindo Grade IV complication. Preop CT planning, LAMPOON, and septal ablation when indicated may help to reduce this risk [26].

#### 4.4.4. Valve Migration

Valve migration is a serious complication affecting TMVR patients [5]. This occurs when the prosthetic valve becomes dislodged from its intended position and may embolize into the left atrium, ventricle, or systemic circulation [20]. This event can lead to acute mitral regurgitation, obstruction of cardiac inflow or outflow, and hemodynamic instability and may present immediate life‐threatening risks. Postdeployment, migration is frequently the result of inadequate anchoring, undersizing, or dislodgement resulting from dynamic cardiac forces [20]. Transapical TMVR allows more direct and controlled deployment within the LV, potentially reducing malposition risk, but the more invasive nature of this approach may introduce other complications like apical trauma that could destabilize the valve [18]. Transseptal TMVR, although less invasive and performed via femoral venous access, may have a higher susceptibility to migration in certain anatomies because device manipulation occurs through a longer catheter pathway, making precise positioning more challenging [11]. Valve migration is classified as a Clavien–Dindo Grade IV event. This complication underscores the vital necessity of exact, careful device positioning, confirming placement with imaging before release.

#### 4.4.5. Postoperative Bleeding

In patients who underwent either TMVR or TEER, postoperative major bleeding necessitating transfusion occurred with greater incidence in the TMVR population. This discovery aligns with the procedure′s nature, with TMVR being inherently more invasive. TMVR often necessitates larger sheath sizes, more extensive vascular access, and greater manipulation within cardiac structures. TMVR is often done using a transapical approach, which necessitates surgical access to the left ventricular apex through a small thoracotomy [15]. This access site carries a greater risk of bleeding, both during and after surgery, because of direct injury to the myocardium, entry into the pericardium, and disruption of tissue with a high density of blood vessels [15]. Even when performed via transseptal access, TMVR involves large‐caliber delivery systems that can increase the risk of access site complications, retroperitoneal hemorrhage, and septal hematomas [15].

Clinically significant major bleeding events necessitating transfusion are categorized as Clavien–Dindo Grade II. Recovery, ambulation, and functional status may all be negatively impacted by even minor blood loss or transfusion in elderly or frail patients, who make up a significant portion of individuals undergoing mitral interventions.

The difference in bleeding rates between TMVR and TEER highlights the necessity of personalized procedural planning and thorough preoperative evaluation of bleeding risk. Such planning may include strategies like careful anticoagulation management, hemostatic closure, and transfusion protocols [15].

#### 4.4.6. Ischemic Cardiomyopathy and Myocardial Infarction (MI)

Postoperative MI was found to be significantly higher in TMVR in comparison to TEER patients. Although it does not happen often, MI after TMVR is a grave complication that arises from multiple factors. TMVR typically necessitates either transapical access or intricate transseptal delivery systems that require considerable handling of cardiac structures [25]. Transapical access, being more invasive, can directly traumatize the myocardium, increase local ischemic stress, and contribute to perioperative hemodynamic instability, potentially raising short‐term MI risk [11, 25].

Despite its rare occurrence, reports of MI in this context are significant due to the high morbidity and mortality linked to myocardial injury in structurally compromised hearts. Complications that may arise after MI include arrhythmias, low cardiac output syndrome, and extended stays in the intensive care unit (ICU) [25]. This finding highlights the significance of preprocedural coronary assessment, particularly for TMVR candidates [25]. It may also emphasize the use of hybrid management strategies, such as staged percutaneous coronary intervention before valve replacement in patients at high risk [25].

Two patients in the TEER group experienced ischemic cardiomyopathy that was unique to this group [13]. Although the overall incidence was low, the occurrence or advancement of ischemic cardiomyopathy after TEER prompts significant inquiries regarding long‐term myocardial remodeling and residual ischemia in this group of patients [25]. TEER is intended to relieve mitral regurgitation without replacing the valve or making significant changes to chamber geometry. However, in patients who have pre‐existing CAD or subclinical ischemia, correcting MR may uncover or worsen underlying left ventricular dysfunction [25].

### 4.5. Hemodynamic Outcomes

**Figure 6:**
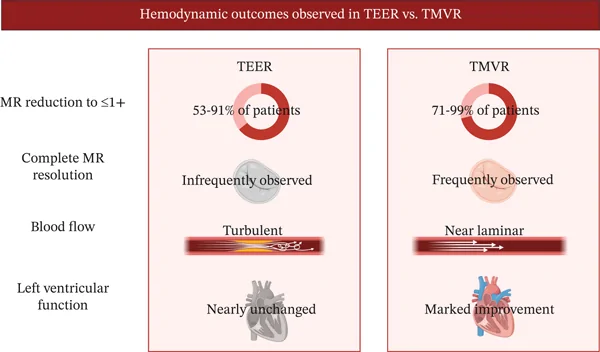
Hemodynamic outcomes observed in TEER versus TMVR. Created in BioRender (Basson, M. [2025]; https://BioRender.com/ao79oia).

TMVR more consistently demonstrated superior efficiency in reducing MR compared with TEER. TMVR was found to reduce MR to either none or mild mitral regurgitation across all studies, but TEER had more variable results across the studies. Between the transseptal and transapical approaches, whereas these access routes primarily influence procedural feasibility, safety, and risk of complications rather than direct hemodynamic efficacy, patients selected for transseptal TMVR may experience lower procedural morbidity, which can indirectly contribute to better functional recovery and ventricular remodeling outcomes [18]. Whereas in TEER, MR was reduced to ≤ 1+ in fewer cases, and more patients had moderate residual MR remaining. This difference is in part due to the nature of the valve implantation that accompanies TMVR, which offers a more robust and uniform reduction of MR. In TEER, the variable results are seen as the procedure relies on each patient′s individual valve anatomy, leading to less predictable outcomes. TMVR also demonstrated better LV function and preserved normal laminar flow patterns, unlike TEER, which distorted mitral valve anatomy, altering the LV and ultimately creating turbulent flow [6]. TMVR was associated with both LV and RV remodeling, ultimately showing a greater reduction in left ventricular end‐diastolic volume index (LVEDVI) and left ventricular end‐systolic volume index (LVESVI) [10]. With a comparable baseline LVEDVI of 81 ± 43 for TEER and 88 ± 24 for TMVR, only TMVR showed a significant reduction at 3 months of 70 ± 40, whereas TEER remained nearly unchanged at 81 ± 42 [10]. Similar results occurred for LVESVI which had a baseline of 51 ± 34 for TEER and 54 ± 20 for TMVR, and again, only a significant reduction at 3 months for TMVR to 48 ± 35 and mostly unchanged for TEER at 51 ± 35 [10]. These findings suggest that the increased ventricular functionality associated with TMVR is due to the technique′s ability to address the mechanical dysfunction at the root of the cause, whereas TEER simply alters the leaflets, not having much effect on ventricular filling and contractility (Figure 6). Additionally, the type of MR, primary versus atrial or ventricular functional, may influence the hemodynamic response, as TMVR can more effectively correct regurgitation caused by extensive leaflet or ventricular pathology, whereas TEER outcomes may be more favorable in cases with preserved ventricular function and suitable leaflet anatomy, such as primary MR or AFMR [16].

### 4.6. Long‐Term Clinical Outcome

One study reported that TEER had a lower reintervention rate (1.1%) than TMVR (1.9%) when comparing the 30‐day reintervention rate [12]. Reintervention rate refers to the number of patients who must undergo an additional procedure or surgical reintervention after the initial therapy, typically due to device failure, complications, or severe residual regurgitation. This increased reintervention rate may be attributed to the complexity associated with TMVR. With its more invasive nature and higher procedural complications, the risk for early reparative intervention is increased. Despite the initial reoperation rate, TMVR appears to be a more durable long‐term solution. Two case reports included in this review examined patients with failed TEER who responded to secondary TMVR, demonstrating TMVR as a better long‐term solution [8, 9]. These cases address the favorable short‐term outcomes of TEER due to fewer early complications; the longevity that is associated with TMVR remains superior. Despite the advantages and disadvantages of each method, there is still more research that needs to be conducted to speak to the long‐term effects of these procedures.

### 4.7. Mortality

It is important to recognize that TEER and TMVR are often used in different clinical scenarios and patient populations. Therefore, the differences observed between these procedures likely reflect, at least in part, variations in patient selection and baseline risk profiles rather than the inherent superiority of one intervention over the other. TMVR was associated with a higher mortality risk than TEER in all the studies except for one measure, the 1‐year outcome in the article by Mack et al. [12]. The increased 30‐day mortality rates for TMVR can be associated with the higher procedural risks and riskier patient population associated with the technique. Transseptal TMVR, being less invasive, may reduce early procedural risk, but still carries higher complexity than TEER, potentially influencing 30‐day mortality. Our overall results indicate that TMVR was associated with higher 30‐day mortality. However, the pooled meta‐analysis demonstrated no statistically significant difference in 1‐year mortality between TMVR and TEER. Other studies included only in‐hospital mortality rates, which they found to be 8.1% in TMVR and 1.9% in TEER [13]. Additionally, one of the studies reported procedural mortality to be 1.7% and 0.5% for TMVR and TEER, respectively, indicating the increased surgical risk for patients undergoing TMVR [11].

### 4.8. Limitations

This systematic review is subject to several limitations that must be acknowledged. First, the majority of the included studies were observational and retrospective in nature, which inherently introduces selection bias and limits the ability to establish causal relationships. Additionally, the review process involved screening and filtering a large volume of articles, and human error must be accounted for as a result. Another limitation is that the review protocol was not prospectively registered, which may increase the risk of methodological deviations during the review process.

There also remains a significant gap in the literature directly comparing TMVR and TEER. Only a small number of studies met the inclusion criteria that required direct comparison of both techniques within the same patient population. This limited the overall scope of comparative analysis and may reduce the generalizability of findings. Additionally, research for these techniques lacks long‐term outcomes and is generally only available as 1‐year outcomes. In order to more accurately compare these methods, researchers must view the outcomes of patients for 5‐ or 10‐year outcomes.

Even though TMVR and TEER are being compared directly, indication bias can be a critical confounding factor. This can be attributed to the fact that patients with higher risk, more complex anatomy, prior failed procedures, or higher baseline surgical risk are more inclined to undergo TMVR. This could have affected the results for complications and mortality when compared with TEER. This introduces indication bias, and the results should be interpreted with caution. This limitation is consistent with the overall serious risk of bias identified using the ROBINS‐I tool.

Lastly, many studies lacked standardized reporting of outcomes, and several important clinical variables, such as precise anatomical criteria, patient comorbidities, transapical versus transseptal TMVR outcomes, or long‐term follow‐up data, were inconsistently reported. In addition, the heterogeneity among the studies included limited our ability to perform quantitative analyses for several complications and outcomes. Therefore, the findings of this review should be interpreted with caution. Further prospective studies and randomized controlled trials are needed to better compare TEER and TMVR across different patient populations and clinical settings.

## 5. Conclusion

With the fast‐evolving field of transcatheter mitral interventions, there is a clear shift in the need for a less invasive yet durable approach to managing mitral regurgitation. As MR becomes more prevalent, particularly with the aging and high‐risk populations, techniques like TMVR and TEER become more critical alternatives to surgical repair or medical therapy. Open‐heart surgery for mitral valve repair or replacement remains the standard of care for patients with a low surgical risk who have suitable anatomy. Additionally, medical therapy or observation is reserved for patients who are asymptomatic and have mild MR with preserved LV function, or for patients who are at a high procedural/surgical risk due to pre‐existing medical conditions. TEER demonstrated a more favorable short‐term safety profile, including lower 30‐day mortality and reoperation rates; however, it is a less effective option for reducing MR, leaving higher grade residual MR in many of its patients. TEER is also reserved for patients with suitable mitral valve anatomy and may not be a generalized operation available to all patients. TMVR proved to be a more complete and superior option for reducing MR, especially when patients were deemed at higher procedural risk. Nevertheless, these findings should be interpreted with caution, as patient selection differences between TMVR and TEER may introduce indication bias and influence the observed outcome. Although these advances are compelling, further research and randomized comparative trials are necessary to further understand the long‐term clinical outcomes of each technique. There remains a need to understand the indications more clearly for each procedure in order to provide the safest and most efficacious solution to each individual MR patient.

## Author Contributions

Rama A. Safadi: conceptualization, methodology, investigation, formal analysis, data curation, validation, writing—original draft, writing—review and editing, and visualization; Alaa Dean A. Esmail: conceptualization, methodology, investigation, formal analysis, data curation, validation, writing—original draft, writing—review and editing, and visualization; Lauren E. DeProfio: conceptualization, methodology, investigation, formal analysis, data curation, validation, writing—original draft, writing—review and editing, and visualization; Ahmed Adham R. Elsayed: conceptualization, methodology, writing—original draft, writing—review and editing, and visualization; Marc D. Basson: conceptualization, methodology, writing—original draft, writing—review and editing, visualization, and supervision. Rama A. Safadi, Alaa Dean A. Esmail, and Lauren E. DeProfio are considered co‐first authors.

## Funding

No funding was received for this manuscript.

## Conflicts of Interest

The authors declare no conflicts of interest.

## Supporting information


**Supporting Information** Additional supporting information can be found online in the Supporting Information section. Supporting Information. Table S1: Search strategy. Table S2: CARE guidelines. Table S3: JBI Critical Appraisal Checklist.

## Data Availability

Data sharing is not applicable, as no new data were collected for this study. All analyses and aggregated calculations are based on previously published data, which are cited within the manuscript.
