# Supplementary material for: Comparing Transcatheter Mitral Valve Replacement to Transcatheter Edge‐to‐Edge Repair in Mitral Regurgitation: A Systematic Review
Source: Cardiovasc Ther. 2026 Sep 26;2026:7522199. doi: 10.1155/cdr/7522199 (PMC13615515; doi:10.1155/cdr/7522199)
Supplement: Supplementary file 1 [file CDR-2026-7522199-s001.docx]

Supplementary Table 1. Search Strategy

| Search strategy | ● PubMed | ((Transcatheter Mitral Valve Replacement OR Catheter-Based Mitral Valve Replacement OR Minimally Invasive Mitral Valve Replacement OR Percutaneous Mitral Valve Implantation OR Endovascular Mitral Valve Replacement OR Non-Surgical Mitral Valve Replacement OR Interventional Mitral Valve Replacement OR TMVR) AND (Transcatheter Edge-to-Edge Repair OR TEER OR Percutaneous Edge-to-Edge Repair OR Endovascular Edge-to-Edge Repair)) AND (Mitral Valve Insufficiency OR "Mitral Valve Insufficiency"[Mesh] OR Mitral valve regurgitation) |
| --- | --- | --- |
|  | - Cochrane - VHL - Web of Science | (Transcatheter Mitral Valve Replacement OR Catheter-Based Mitral Valve Replacement OR Minimally Invasive Mitral Valve Replacement OR Percutaneous Mitral Valve Implantation OR Endovascular Mitral Valve Replacement OR Non-Surgical Mitral Valve Replacement OR Interventional Mitral Valve Replacement OR TMVR) AND (Transcatheter Edge-to-Edge Repair OR TEER OR Percutaneous Edge-to-Edge Repair OR Endovascular Edge-to-Edge Repair) AND (Mitral Valve Insufficiency OR Mitral Valve Regurgitation OR Mitral Incompetence OR Mitral Regurgitation OR Mitral Insufficiency OR Mitral valve incompetence OR MR OR incompetent mitral valve OR backflow through mitral valve OR leaky mitral valve OR mitral valve leakage OR mitral valve dysfunction OR mitral valve dysfunction with regurgitation)  ("Transcatheter Mitral Valve Replacement" OR "Catheter-Based Mitral Valve Replacement" OR "Minimally Invasive Mitral Valve Replacement" OR "Percutaneous Mitral Valve Implantation" OR "Percutaneous Mitral Valve Replacement" OR "Endovascular Mitral Valve Replacement" OR "Non-Surgical Mitral Valve Replacement" OR "Interventional Mitral Valve Replacement" OR TMVR) AND ("Transcatheter Edge-to-Edge Repair" OR TEER OR “Percutaneous Edge-to-Edge Repair” OR “Endovascular Edge-to-Edge Repair” OR “Catheter Based Edge-to-Edge Repair”) AND (“Mitral Valve Insufficiency” OR “Mitral Valve Regurgitation” OR “Mitral Incompetence” OR “Mitral Regurgitation” OR “Mitral Insufficiency”)  ((ALL=(Transcatheter Mitral Valve Replacement OR Catheter-Based Mitral Valve Replacement OR Minimally Invasive Mitral Valve Replacement OR Percutaneous Mitral Valve Implantation OR Endovascular Mitral Valve Replacement OR Non-Surgical Mitral Valve Replacement OR Interventional Mitral Valve Replacement OR TMVR)) AND ALL=((Transcatheter Edge-to-Edge Repair OR TEER OR Percutaneous Edge-to-Edge Repair OR Endovascular Edge-to-Edge Repair))) AND ALL=((Mitral Valve Insufficiency OR Mitral Valve Regurgitation OR Mitral Incompetence OR Mitral Regurgitation OR Mitral Insufficiency OR Mitral Valve Incompetence OR MR OR Incompetent Mitral Valve OR Backflow Through Mitral Valve OR Leaky Mitral Valve OR Mitral Valve Leakage OR Mitral Valve Dysfunction OR Mitral Valve Dysfunction with Regurgitation)) |
| Date initiated | 1/12/2025 | |
| Initial findings | Total = 1162   - PubMed: 578 - Cochrane: 42 - VHL:64 - WOS: 478 | |
| Removed before screening (Duplicates) | Excluded= 448  Included= 714  Confirmed: 1/13/25 | |

Supplementary Table 2. CARE Guidelines

Supplementary Table 3. JBI Critical Appraisal Checklist
